# Acute Bilateral Renal Infarction: An Unusual Complication After Mitral Valve Replacement Surgery

**DOI:** 10.7759/cureus.14037

**Published:** 2021-03-22

**Authors:** Amal Haoudar, Jihane Ziati, Said Makani, Khalid Agrad, Chafik El Kettani

**Affiliations:** 1 Anesthesia and Critical Care, Cheikh Khalifa International University Hospital, Mohammed VI University of Health Sciences, Casablanca, MAR; 2 Cardiac Surgery, Cheikh Khalifa International University Hospital, Mohammed VI University of Health Sciences, Casablanca, MAR

**Keywords:** renal infarction, mechanical mitral prosthesis, acute kidney injury, chronic kidney disease, atrial flutter

## Abstract

Bilateral renal infarction is an uncommon clinical condition that is frequently misdiagnosed. Its various mechanisms mainly include thrombotic and embolic. We report the case of a 51-year-old Moroccan woman, who presented with acute bilateral renal infarction three days following mitral valve replacement surgery of probable embolic origin despite curative heparin. Her renal function did not improve, and the patient developed chronic renal failure. Diagnosing bilateral renal infarction is challenging, especially in the postoperative period of mechanical mitral valve replacement surgery. Curative heparin therapy does not totally protect (100%) against this serious complication. This case study aimed to raise awareness of this rare clinical condition after cardiac surgery.

## Introduction

Bilateral renal infarction is an uncommon clinical condition with various mechanisms, mainly thrombotic and embolic [1]. Several studies have shown that the incidence among all emergency admissions was as low as 0.007% [2]. Bilateral renal infarction was reportedly found in patients with dissecting aneurysms of the aorta, lupus, vasculitis, sickle cell disease, fibromuscular dysplasia of the renal arteries, and trauma [3].

Renal artery occlusion causes permanent renal parenchymal damage and renal failure. It should be considered in patients at high risk for embolic events (atrial fibrillation, left atrial dilatation, mechanical mitral prosthesis) presenting with inexplicable acute abdominal pain associated with hematuria and acute kidney injury [1,4].

The bilateral occurrence of renal infarction in the postoperative period of mitral valve replacement surgery is an exceptional finding, and to our knowledge, no cases have previously been reported in the literature. We report a case of bilateral renal infarction, which caused acute kidney injury leading to chronic kidney disease during the early postoperative period of mitral valve replacement surgery, despite effective anticoagulation therapy.

## Case presentation

We report the case of a 51-year-old Moroccan woman admitted to the cardiovascular intensive care unit after mitral valve replacement surgery. Her medical history was significant for organic mitral disease (mild mitral stenosis and severe regurgitation) with preserved left ventricular function, enlarged left atrium, atrial flutter, irritable bowel, and depression. She had been taking acenocoumarol (4 mg) for the last two years to prevent thromboembolic events. The surgical procedure consisted of valvular replacement surgery with a mechanical prosthesis. No thrombus was detected in the left atrium during the perioperative period. During the weaning of the cardiopulmonary bypass (73 min), the patient exhibited supraventricular tachycardia, which was reduced using amiodarone.

During the postoperative period, the hemodynamics were stable without any inotropic or vasopressor agents, and the cardiac rhythm was regular. In the postoperative stage, the patient received fluids, an intravenous curative dose of unfractionated heparin (UFH), and antalgics. The blood test results are presented in Table 1. Three days later, the patient reported generalized abdominal pain that spread to the flanks with hematuria, constipation, and vomiting. Physical examination showed the following vital signs: body temperature, 37.2°C; blood pressure, 110/78 mmHg; heart rate, 77 beats per minute; respiratory rate, 22 breaths per minute; and swollen abdomen with a slight stiffness in the renal angles. The rest of the examination results were normal.

**Table 1:** Laboratory finding before and after surgery D: Day D0: The day of the surgery

	D-1	D0	D+1	D+2	D+3	D+4	D+5	D+6	D+7	D+8	D+15
Blood leukocyte count, 10ᶟ/mmᶟ	8500	-	16200	-	17400	-	-	14620	13640	-	-
Platelet count, 10ᶟ/mm	279000	-	165000	-	135000	-	-	339000	340000	-	-
Hemoglobin, g/dL	13.2	-	11.2	-	8.9	-		11.7	11.3		
Blood urea nitrogen, g/L	0.37	-	0.26		0.63	0,87	1.20	1.54	1.69	2.03	2.29
Creatinine, mg/L	8.66	-	7.05		30.87	45.37	56.16	64.04	70.12	77.57	92.97
C-reactive protein level, mg/L	1.4	-	65	-	261.40	-	-	232	-	-	66
Activated partial thromboplastin time sec ratio	1	-	3.17	4.57	3.43	-	-	-	-	-	-
International normalized ratio	-	-	-	-	-	4.75	3.23	3.26	-	-	5.43
Serum lipase	-	-	-		24	-	-	-	-	-	-
Procalcitonin, ng/ml	-	-	-	-	4.54	-	-	-	-	-	-
Lactico deshdrogenase, UI/L	-	-	-	-	-	827	-	-	-	-	-

**Figure 1:**
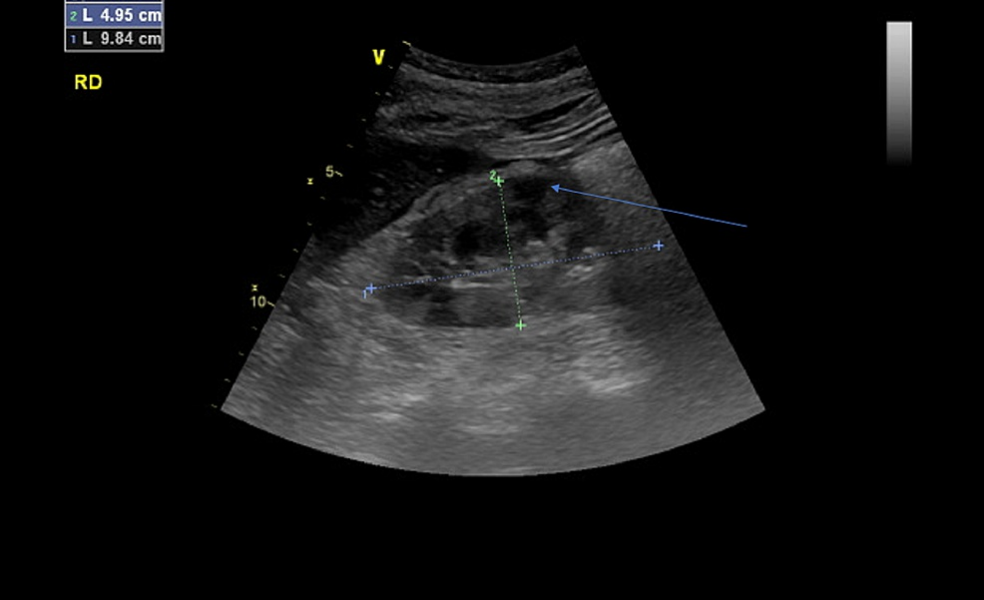
Duplex Doppler ultrasound showed aspects of multifocal cortical renal infarctions in the right kidney (arrow)

Pyelonephritis or mesenteric ischemia was strongly suspected as the cause of her acute pain. The workup revealed high serum creatinine levels, high lactate dehydrogenase (LDH) levels, high leukocytosis, and high C-reactive protein (CRP) levels. Urine analysis showed macroscopic hematuria and aseptic leukocytosis. An abdominal CT scan without contrast showed fecal impaction and infiltration of perirenal fat. Duplex Doppler ultrasound showed aspects of bilateral multifocal cortical renal infarctions (Figure 1), which suggested arterial thromboembolic disease.

The aorta and renal arteries were permeable with no atherosclerotic plaques. Both the renal vein and inferior vena cava were permeable. Transthoracic and transesophageal echocardiography showed neither intracardiac nor valvular prosthesis thrombus or endocarditis vegetation. The estimated left ventricular ejection fraction was 38% with elevated left ventricular filling pressure. The systolic pulmonary arterial pressure was approximately 46 mmHg.

We concluded that it was a bilateral renal infarction of probable embolic origin, despite curative heparin. A progressive increase was observed in serum creatinine levels up to 70 mg/L after four days. The urine output up to now was preserved. One month later, the creatinine level increased to 95 mg/L, and the patient was diagnosed with chronic kidney disease.

## Discussion

We report a case of bilateral renal infarction following mitral valve replacement surgery in a 51-year-old woman with subsequent acute kidney injury and chronic kidney failure.

Even in the postoperative period of cardiac surgery, risk factors for thromboembolic events, such as arrhythmia, ventricular dysfunction, low cardiac output, left atrial dilatation, and septic emboli from endocarditis, are frequent. The occurrence of bilateral renal infarction remains exceptional. Several studies have described embolic complications, such as ischemic stroke and limb ischemia, while acute bilateral renal infarction has never been reported. The substantial risk of thromboembolic events following mechanical mitral valve replacement can occur even when bridging anticoagulation is initiated earlier in the postoperative period. In an observational study on patients who received intravenous UFH following mechanical valve replacement with early effective anticoagulation, the rate of clinical thromboembolic events within 30 days was 5% for mitral valve replacement (alone or combined with aortic valve replacement) [5]. The risk factors for thromboembolism included the temporary withdrawal of anticoagulation for pacemaker implantation, heparin-induced thrombocytopenia, and diabetes mellitus. To the best of our knowledge, no cases of bilateral renal infarction have been reported.

Acute bilateral renal infarction is an extremely rare condition that is frequently misdiagnosed, especially in the context of postoperative cardiac surgery. Unless there is a high index of suspicion for it, renal infarction is difficult to detect because of its non-specific signs and symptoms [6]. Renal infarction mainly manifests as non-specific clinical signs. Flank pain is sometimes associated with hematuria. In this context, these two signs could be manifestations of other complications (paralytic ileus, upper urinary infection, and mesenteric ischemia). The occurrence of acute kidney injury with flank pain and hematuria outside of the context of cardiac surgery is alarming. However, in the context of patient-administered anticoagulants for mitral valve replacement surgery, hematuria may be the result of excessive anticoagulation or urinary tract infection. In acute kidney injury, several risk factors such as low cardiac output, myocardial dysfunction, anemia, and hypovolemia may precipitate its occurrence. This makes diagnosis even more difficult. Moreover, renal infarction is an alert state since cerebral and peripheral embolisms can occur at any time [7]. During the postoperative period of mechanical replacement valve surgery, patients receive effective anticoagulation with close control to prevent thromboembolic events. The risk of thromboembolism increases in patients with atrial fibrillation and mechanical valves. Furthermore, this risk increases with left atrial enlargement, regardless of the degree of anticoagulation. Consequently, bilateral renal infarction causes acute kidney injury (AKI) and chronic kidney disease (CKD). Their incidence is heterogeneous in the literature (AKI incidence of 8-64% and CKD incidence of 6-32.5%). AKI is a major risk factor for the development of CKD, and this risk may persist away from the acute episode even after full recovery and despite mild impairment of the renal function. Presently, the progression from AKI to CKD has not yet been clarified. Suggestively, inappropriate vascular, interstitial, and tubular regeneration may be resulting in renal fibrosis responsible for the progression to renal failure [8]. AKI after cardiac surgery is independently associated with a significant increase in morbidity, mortality, and healthcare costs [9]. It is crucial to pay attention to and recommend long-term follow-up of patients with an initial AKI episode. High CRP levels were an independent risk factor for AKI after renal infarction. It is a good indicator of loss of kidney function since the level of CRP elevation is correlated with the severity of renal parenchyma aggression. When suspecting renal infarction, a CT scan with contrast remains the gold standard examination to establish the diagnosis. A renal echo-Doppler was chosen for our patient in order to prevent worsening of renal function. The therapeutic management is based on anticoagulation, thrombolysis, and renal replacement therapy, if required. In this case, the patient was under effective anticoagulants, and thrombolysis was not performed because she had contraindications. The prognosis of bilateral renal infarction is uncertain. In this case, the patient developed CKD.

## Conclusions

In conclusion, the diagnosis of bilateral renal infarction is challenging in patients in the postoperative period of mechanical mitral valve replacement surgery despite effective anticoagulation therapy. The renal function did not improve, and the patient had developed CKD.

Acute bilateral renal infarction is a rare complication with a poor prognosis that should be considered in patients presenting with flank pain and AKI. Patients should undergo thorough biological monitoring of etiological factors and receive prompt treatment.

Curative heparin therapy did not completely protect (100%) against this serious complication.
